# Periodic Limbic Movement Disorder during Sleep as Diabetes-Related Syndrome? A Polysomnographic Study

**DOI:** 10.5402/2011/246157

**Published:** 2011-08-07

**Authors:** M. Rizzi, M. Barrella, G. D. Kotzalidis, M. Bevilacqua

**Affiliations:** ^1^Institute of Pneumology, Luigi Sacco Hospital, Giovanni Battista Grassi Street 74, 20157 Milan, Italy; ^2^Institute of Endocrinology, Luigi Sacco Hospital, Giovanni Battista Grassi Street 74, 20157 Milan, Italy; ^3^Department of Neurosciences, Mental Health and Sensory Organs (NESMOS), Sapienza University, School of Medicine and Psychology, Sant'Andrea Hospital, Via di Grottarossa 1035-1039, 00189 Rome, Italy

## Abstract

*Introduction*. Periodic limb movements during sleep (PLMs) is common in the elderly. When quality-of-life drops due to sleep disturbances, we speak about periodic limb movement disorder during sleep (PLMD). Another similar disorder, restless legs syndrome (RLS), is considered to be related to diabetes; RLS and PLMDs are genetically related. Our aim was to detect PLMDs in a population of diabetic patients and identify them as possible hallmarks of these autonomic disorders. *Material and Methods*. We selected 41 type-2 diabetics with no sleep comorbidity, and compared them with 38 healthy matched volunteers. All participants underwent the Epworth Sleepiness Scale (ESS) and polysomnography (PSG). A periodic limb movement (PLM) index >5, that is, the higher number of PLMs/sleep hour for the entire night, was considered as abnormal. *Results*. Diabetics showed lower sleep efficiency than controls on the ESS, lower proportions of REM and non-REM sleep, and higher arousal and PLM indexes, as assessed through PSG. PLMDs were diagnosed in 13 of 41 diabetic patients (31%); the latter showed lower sleep efficiency, lower non-REM slow-wave sleep, and increased arousal and PLM indexes. *Conclusion*. The relationship between PLMs-related sleep fragmentation and endocrine carbohydrate metabolism regulation might be casual or genetically determined. This deserves further investigations.

## 1. Introduction

Until recently, it was believed that sleep was important primarily for restoring brain functions. However, there is increasing evidence that sleep also modulates the metabolic, endocrine and cardiovascular systems [1]. Diabetes is predicted to affect 221 million people worldwide by the end of 2010; this would amount to a 46% increase from 2000 [2]. In industrialized countries, chronic sleep loss is rapidly increasing along with diabetes, and already affects about 45% of all adults [3]. Furthermore, alterations in the control of blood sugar may gradually lead to serious dysregulation of sleep control and vice versa. In fact, quite intriguingly, a dramatic increase in the incidence of diabetes and obesity appears to develop simultaneously with decreased self-reported sleep duration; this may indicate a close relationship between diabetes and sleep disorder [4, 5]. In fact, data from the Sleep Heart Health Study showed that people with diabetes had less time spent on REM sleep and more breathing episodes than people without diabetes [6]. In type 2 diabetes, sleep disturbances are believed to be common and have been attributed to impaired glucose metabolism and general physical distress [7]. An association between diabetes and restless legs syndrome (RLS) has also been reported [8–10], but other investigations did not confirm the evidence [11]. RLS is characterized by (a) a drive to move the legs often, accompanied by abnormal leg sensations, (b) symptom worsening at rest, that is, sitting, (c) partial or temporary relief by activity, at least as long as the activity continues, and (d) worsening of the symptoms later in the day or night [12].The vast majority (90%) of patients have stereotyped repetitive dorsiflexions of the big toe and foot once asleep, a condition known as periodic limb movements during sleep (PLMs) [13, 14]. Although RLS and PLMs can occur independently, their frequent association suggests a common etiology. This has been confirmed recently by the discovery of a common gene variant (BTBD9) that confers susceptibility to both PLMs and RLS [15, 16]. It has been shown that the prevalence of PLMs is correlated with age; it is found in 5% of normal subjects aged between 30 and 50 years, in 29% of subjects over 50 years and in 44% of subjects aged 65 and older [17]. PLMs have been linked to sleep fragmentation [18] and corre1ated with impaired subjective sleep quality [19]. Various polysomnographic studies indicate that PLMs are often associated with electroencephalographic arousal [20–23] or with phase A of the cyclic alternating pattern [24], suggesting a disruption of sleep continuity. When PLMs cause disturbed sleep, inducing insomnia or daytime sleepiness, a periodic limb movement disorder during sleep (PLMDs) is diagnosed [25]. We aimed at investigating the presence of sleep disturbance due to PLMs in a cohort of diabetes type 2 patients.

## 2. Patients and Methods

A group of 45 consecutive patients with type 2 diabetes were compared with 38 healthy, age-, sex-, and body-mass index (BMI-) matched volunteers (Table l). The study was approved by the hospital's medical ethics committee, and informed consent was obtained from all participants. Exclusion criteria comprised neurologic or psychiatric disorders, tobacco, alcohol or drug abuse, history of sleep apnea syndrome or obesity, chronic renal failure or uremia, polyneuropathy, rheumatoid arthritis, anemia, hypothyroidism, chronic obstructive pulmonary disease, heart disease, liver cirrhosis, and B12 deficiency. All the study participants, before polysomnography (PSG) were asked to identify the most likely factors contributing to sleep complaints and filled-out the Epworth Sleepiness Scale (ESS) [26]. The ESS is an 8-item self-rated questionnaire measuring the general level of daytime sleepiness, each rated according to a 0–3 Likert scale. It can vary from 0 to 24; the “clinically” normal range of the ESS score is 0 to 10 with a normal statistical distribution; scores of 11-12 are borderline, and higher scores are probably pathological, needing expert consultation. PSG is the study of sleep by means of the electroencephalogram (EEG), electro-oculogram (EOG), electrocardiogram (ECG), and submental and tibial electromyograms (EMG). In addition, recordings involve the measurement of oxygen saturation, nasal and oral airflow, and thoracic and abdominal respiratory movements [27]. The PSG was performed in our sleep laboratory, a sound-attenuated room with temperature control, using a computer-assisted device (Alice 3; Healthdyne, Marietta OH). EEG, EOG, ECG, and EMG were recorded with surface electrodes using standard techniques [27]. Airflow and ventilatory efforts were recorded through nasal oral thermocouples and thoracic and abdominal belts with inbuilt piezoelectrodes, respectively. Oxyhemoglobin saturation was recorded by finger pulse oximetry (Pu1sox-7 MinoIta, Osaka, Japan). The transducers and lead wires allowed normal position changes during sleep. To qualify as PLMs, leg movements had to last 0.5–10 sec, to recur every 5 to 90 sec, and to occur in a series of at least four such movements in a row that are at least 8 *μ*V in amplitude [27]; periodic movements with close temporal relation to apnoea or hypopnoea were not interpreted as PLMs. A PLMs index (numbers of PLMs per hour of sleep) greater than 5 for the entire night of sleep was considered to be pathological. Criteria for a diagnosis of PLMDs included a PLM index of at least 20 per hour and the presence of either excessive daytime sleepiness (ESS score > 6) and a subjective feeling of non-restorative or disturbed sleep [24]. Arousals were scored according to the American Academy of Sleep Medicine criteria [27]. Bedtime and awakening time were at each participant's discretion and the PSG was terminated after final awakenings. To avoid the first night effect each subject spent 2 nights in the sleep laboratory. Only data recorded during the second night were evaluated.

## 3. Statistical Analysis

Data were reported as means and standard deviations. Statistical analysis of the anthropometric data and polysomnographic recordings was performed using unpaired Student's *t*-test. Pearson's Chi-square goodness of fit was used for other comparisons of means and proportion. The Mann-Whitney *U*-test and Spearman's rank correlation were used as appropriate. The level of significance was set at *P* < 0.05. All statistical analyses were performed using the statistical package SPSSV 6.l (SPSS, Inc., Chicago, IL).

## 4. Results

Four out of 45 patients (9%) were excluded from the study because they were affected by the sleep apnea syndrome (Apnea-Hypopnea index at least 10 per hour).  Table 1 shows the anthropometric data of patients with type 2 Diabetes and healthy controls, and the most common sleep complaints, that is, nocturnal awakening (62%), chronic poor sleep (48%) and sleepiness (50%) (*P* < 0.01 versus controls). Moreover patients with diabetes scored higher on the ESS, compared to controls (*P* < 0.05). The sleep values (Table 2) show that patients with diabetes had lower sleep efficiency (sleep time/time in bed) than controls (*P* < 0.01); the percentage of REM sleep and non-REM slow wave sleep were significantly lower in diabetics patients (*P* < 0.01); arousal index and periodic limb movement index were significantly greater in diabetic patients (*P* < 0.01). A significant correlation with the periodic limb movement index was documented for sleep efficiency, arousal index and ESS score (*r* = −0.56; *P* < 0.01; *r* = 0.78; *P* < 0.01; *r* = 0.93; *P* < 0.001, resp.; Table 3). In 13 of 41 diabetic patients (31%) PLMDs was diagnosed; in these patients sleep efficiency and non-REM slow wave sleep were significantly lower (*P* < 0.1) and both arousal and periodic limb movement indexes were significantly increased (*P* < 0.01) compared to diabetic patients without PLMDs (Table 4).

## 5. Discussion

A large proportion of our diabetes patients reported having poor sleep quality; in particular the prevalence of PLMs, in our population of type II diabetes patients is greater (85%) in comparison with the general populations of the same age (44%). These findings were consistent with previous studies [9]. The nonrestorative sleep of these patients cannot be directly related to a specific disruption of sleep architecture; however, the diabetes type 2 patients are more easily identified because they have pathological PLMs indexes. Indeed, sleep fragmentation linked to PLMs causes daytime sleepiness. The analysis of the subgroups of diabetes patients with PLMDs, as shown in Table 4, showed a marked disruption of sleep architecture, a greater arousal index, and higher ESS score with respect to diabetes patients with only PLMs; we consider this as the most important finding of this study. In fact, sufficient evidence about bidirectional interactions between sleep, and the endocrine system exists currently; several hormones have the capacity to affect sleep and sleep is a key factor in physiological restoration, with far-reaching medical implications. Therefore, although the primary function of sleep may be cerebral restoration, insufficient sleep has consequences also for peripheral function, that if maintained chronically, could impact endocrine regulation of carbohydrate metabolism, including decreased insulin sensitivity [5]. A partial sleep deprivation (4 hours per night for 6 consecutive nights) may trigger impaired glucose tolerance, higher evening secretion of cortisol, increased sympathetic nervous system activity, and reduced leptin (the appetite suppressant) secretion [28] and might produce a prediabetic state [5]. Guggisberg et al. [25] suggest an important role of the sympathetic system in the generation of PLMs that we can try to incorporate in this model. A sympathetic activation occurs periodically in the setting of the physiologic sleep-wake control; once overcoming a certain threshold, it triggers or facilitates PLMs. Thus, the magnitude and frequency of movements would depend on the magnitude and frequency of the sympathetic oscillation. Previous clinical drug trials have suggested an involvement of the sympathetic nervous system, that is, also involved in the generation of sensory discomfort (i.e., increased vascular tone and decreased blood flow to the extremities), which accompanies PLMs [29]. Previous studies speculated that potentially dysfunctional extrapyramidal motor networks are periodically activated by sympathetic projections and, thus, induce PLMs [30]. In patients with dysfunctional extrapyramidal system due to Parkinson syndrome [31], normal fluctuations in sympathetic activity might be sufficient to trigger PLMs, the autonomic nervous system being merely responsible for the periodicity of the movements. Gallego et al. [32] showed that the dopamine content of diabetic rats was reduced in several areas of the central nervous system, including striatum and midbrain. Based on these considerations, Merlino et al. [33] suggested that RLS in patients affected by type 2 diabetes could be due to the coexistence of decreased dopaminergic control and increased excitatory nociceptive input, the latter due to peripheral neuropathy and both PLNs and RLS involved in the pathogenesis dopaminergic deficit.

## 6. Conclusion

It could be important to detect PLMDs in type 2 diabetes patients, because poor sleep decreases glucose tolerance and insulin sensitivity, further worsening the clinical picture of diabetes and conferring resistance to treatment. Adequately treating PLMs could normalize sleep architecture and efficiency, improving both quality of life and the control of the diabetic condition.

## Figures and Tables

**Table 1:** Demographic and clinical data in 41 type II diabetes patients and 38 healthy controls.

	Type II diabetes patients	Healthy controls	*P*
Age, yrs	63 ± 4	61 ± 5	ns
Sex F/M	23/18	21/17	ns
BMI, Kg/m^2^	27 ± 3	26 ± 5	ns
ESS score	10 ± 2	4 ± 2	0.05
Chronically poor sleep %	48	15	0.01
Nocturnal awakening %	62	20	0.01
Sleepiness (somnolence) %	50	8	0.01

Data presented as mean ± SD, level of significance *P* < 0.05.

BMI: body mass index; ESS: Epworth Sleepiness Scale; ns: not significant, F: female; M: male.

**Table 2:** Sleep values in 41 type II diabetes patients and 38 healthy controls.

	Type II diabetes patients	Healthy controls	*P*
Sleep time, min	307 ± 54	398 ± 41	0.001
Sleep efficiency, %	77 ± 10	89 ± 6	0.01
nREM stage 1, % sleep time	14 ± 5	13 ± 5	ns
nREM stage 2, % sleep time	42 ± 7	40 ± 8	ns
nREM slow wave, % sleep time	13 ± 4	21 ± 6	0.01
REM, % sleep time	9 ± 4	15 ± 3	0.01
AHI, n/h	3 ± 2	3 ± 1	ns
Arousal index, n/h	14 ± 6	5 ± 1	0.01
PLM Index, n/h	22 ± 9	5 ± 1	0.001

Data presented as mean ± SD, level of significance *P* < 0.05.

REM: rapid eye movements, AHI: apnea hypopnea index, PLM: periodic limb movement.

**Table 3:** Spearman's rank correlation (*r*) between periodic limb movement index, and sleep functional data and Epworth Sleepiness Scale score.

Periodic limb movement index versus	*r*	*P*
Sleep efficiency	−0.56	0.0001
ESS score	0.93	0.0001
Total sleep time	−0.67	0.01
Arousal index	0.78	0.0001

Level of significance *P* < 0.05.

**Table 4:** Comparison of sleep-related data in 13 type 2 diabetes patients subdivided according to the presence of periodic limb movement disorder during sleep (PLMDs).

PLMDs	Yes, *n* = 13	No, *n* = 28	*P*
Sleep efficiency, %	71 ± 6	83 ± 4	0.01
Non-REM slow wave, % sleep time	8 ± 5	15 ± 4	0.01
Arousal Index, n/h	17 ± 4	9 ± 3	0.01
PLM Index, n/h	28 ± 7	15 ± 8	0.01

Data presented as mean ± SD; level of significance *P* < 0.05.

The parameters reported are only those which showed a statistically significant intergroup difference.
